# Nitric oxide production rather than oxidative stress and cell death is associated with the onset of coral bleaching in *Pocillopora acuta*

**DOI:** 10.7717/peerj.13321

**Published:** 2022-06-01

**Authors:** Christopher P. Jury, Brian M. Boeing, Henry Trapido-Rosenthal, Ruth D. Gates, Robert J. Toonen

**Affiliations:** 1 Hawaiʻi Institute of Marine Biology, Kāneʻohe, HI, United States; 2 Biology, Chaminade University, Honolulu, HI, United States

**Keywords:** Nitric oxide, Oxidative stress, Coral bleaching, Coral-algal symbiosis, Climate change

## Abstract

Elevated seawater temperatures associated with climate change lead to coral bleaching. While the ultimate causes of bleaching are well understood, the proximate physiological mechanisms underlying the bleaching response are not as well defined. Here we measured nitric oxide synthase activity, oxidative stress, and cell death in algal symbionts (Symbiodinaceae) freshly isolated from the reef-building coral *Pocillopora acuta* collected in the field under natural non-bleaching conditions and from corals experimentally exposed to elevated temperatures. Nitric oxide synthase activity in the algal symbionts was >3 orders of magnitude higher than that of the host and increased dramatically with increasing temperature and time of exposure (up to 72 h), consistent with the onset of bleaching for these corals. Oxidative stress and cell death among the algal symbionts were highest in coral holobionts exposed to intermediate as opposed to maximal temperatures, suggesting that these mechanisms are not proximal triggers for bleaching in this species. Our results point to nitric oxide production by the algal symbionts, rather than symbiont dysfunction, as a more important driver of coral bleaching under acute thermal stress in this coral.

## Introduction

Tropical reef-building corals as well as a variety of other cnidarians, protists, and molluscs, form symbiotic associations with unicellular dinoflagellates of the family Symbiodiniaceae (referred to collectively as symbionts hereafter). The symbionts translocate a substantial fraction of their photosynthetically fixed carbon (photosynthate) to the hosts, which can meet 100–150% of the daily respiratory needs of shallow water corals and 80–90% of their total fixed carbon intake (Muscatine & Cernichiari, 1969; Grottoli, Rodrigues & Palardy, 2006) Symbiont photosynthesis also produces large amounts of O_2_, which supports metabolically demanding activities, such as coral calcification (Colombo-Pallotta, Rodríguez-Román & Iglesias-Prieto, 2010).

Coral bleaching reflects a severe reduction in symbiont densities and/or photosynthetic pigment concentrations and is a physiological response often associated with heat stress (Glynn, 1993; Fitt et al., 2001; Douglas, 2003; Jokiel & Brown, 2004). Bleaching can result in lower host growth rates, interruptions in sexual reproduction, increased incidence of disease, and mortality, with the severity of these responses varying depending on the extent of bleaching and the sensitivity of the coral taxa (Hughes et al., 2003; Hoegh-Guldberg et al., 2007; Johnston et al., 2020). Seawater temperatures around most coral reefs have increased by 0.5–1 °C over the last century and are expected to increase a further 0.5–3.6 °C this century, depending on greenhouse gas emissions scenario (Hoegh-Guldberg, 1999; Donner, 2009; Heron et al., 2016; Lough, Anderson & Hughes, 2018). With the trends in ocean warming, mass coral bleaching is predicted to increase in frequency and severity, potentially endangering the persistence of coral reefs as we know them within the next few decades (Hoegh-Guldberg et al., 2007; Donner, 2009; Frieler et al., 2013; van Hooidonk, Maynard & Planes, 2013).

The endosymbionts of corals are genetically diverse, comprising nine major lineages that are physiologically distinct (Pochon, Putnam & Gates, 2014; LaJeunesse et al., 2018). Differences in taxonomic composition in the symbiosis can manifest in higher or lower thermal tolerance for the host and shifts in the symbiosis may provide an avenue for coral acclimatization to climate change (Baker et al., 2004; Barshis et al., 2013). Some corals also inhabit thermally variable environments and tolerate regular, short-term exposure to temperatures above local bleaching thresholds without undergoing bleaching (Coles, 1975; Barshis et al., 2010). Both acclimatization to the local environment and selection for higher thermal tolerance, with or without shifts in symbiont communities, have been implicated in driving these responses (Warner et al., 2006; Howells et al., 2012; Cunning, Silverstein & Baker, 2015; Bay et al., 2016).

While the ultimate causes of coral bleaching are relatively well understood, the proximate physiological mechanisms that drive bleaching as well as the physiological underpinnings of differential thermal sensitivities among corals are less well understood (Baker, 2003; Hoadley et al., 2015; Grégoire et al., 2017). One mechanism that explains part of the variation in thermal sensitivity is differing lipid composition of the thylakoid membranes of the chloroplasts of symbionts. Thermally sensitive symbionts tend to have lower membrane melting points and distinct membrane composition as compared to more thermally tolerant ones (Tchernov et al., 2004; Díaz-Almeyda et al., 2011). Increased membrane fluidity under elevated temperature physically decouples electron transport between photosystem II (PSII) and proteins associated with the electron transport chain of photosynthesis (Tchernov et al., 2004; Díaz-Almeyda et al., 2011). This decoupling leads to increased production of reactive oxygen species (ROS), damaging the reaction centers of PSII, and leading to oxidative stress as ROS production exceeds the antioxidant capacity of algal and coral cells. Oxidative stress can lead to tissue necrosis and host cell detachment during bleaching (Gates, Baghdasarian & Muscatine, 1992). Symbionts released from some corals in response to high temperature stress, however, remain photosynthetically competent (Ralph, Gademann & Larkum, 2001), meaning that bleaching is not simply a matter of purging damaged algal cells from coral tissues but that other mechanisms are also likely critical for initiating and mediating the bleaching process.

Increased nitric oxide (NO) production has emerged as one potential driver of cnidarian bleaching. NO is produced by nitrate or nitrite reductase and a family of proteins collectively referred to as nitric oxide synthases (NOS). These proteins convert arginine → citruline + NO, with a 1:1 stoichiometry between citruline and NO production. Under elevated temperatures, NO production increases in symbiotic anemones and corals, which is followed by bleaching (Trapido-Rosenthal et al., 2001, 2005; Perez & Weis, 2006; Bouchard & Yamasaki, 2008; Hawkins & Davy, 2013; Hawkins et al., 2014). In addition, bleaching can be induced in symbiotic anemones at normal temperature with the NO donor SNP, and can be inhibited under elevated temperature with the NO scavenger cPTIO, suggesting that elevated NO production plays a key role in the bleaching process (Perez & Weis, 2006).

The purpose of this study was to examine the relative importance of algal symbiont NOS activity, oxidative stress, and cell death during the early stages of bleaching in a reef-building coral. These responses were assessed in samples freshly collected from the field during a non-bleaching period, thereby providing a baseline of natural variability, against which to compare samples produced through a temperature perturbation experiment under controlled conditions.

## Methods

### Study species and collection site

The scleractinian *Pocillopora acuta* (which has historically been misidentified as its sister species, *P. damicornis*, in Hawai‘i and other locations) (Poquita-Du et al., 2017; Johnston, Forsman & Toonen, 2018) was selected for this investigation because it is abundant on the shallow reefs adjacent to the Hawai‘i Institute of Marine Biology (HIMB), broadly distributed in the Pacific, well studied, and an important reef-builder in some parts of its range. Coral branches 3–6 cm in length were collected at 1–2 m depth with bone shears from colonies on the fringing reef adjacent to HIMB under a permit issued by the Department of Land and Natural Resources (Permit Number: SAP 2006-02).

### Field investigation

To contextualize the results of our temperature perturbation experiments (described below) we examined NOS activity, oxidative stress and cell death in symbionts freshly isolated from *P. acuta* collected from the field between June, 2006 and September, 2006. During the summer and early autumn, seawater temperatures around HIMB typically range from 27 to 28 °C, sometimes reach 28–29 °C for up to several weeks, but rarely exceed 29 °C (Jokiel & Brown, 2004; Bahr, Jokiel & Toonen, 2015; Jury & Toonen, 2019). Seawater temperature on the reef during the timeframe of this study was obtained from the National Oceanographic and Atmospheric Administration (NOAA) Tides and Currents website (tidesandcurrents.noaa.gov) for the Moku o Lo‘e climate station located at HIMB.

A single coral branch was collected from each of 1–3 haphazardly selected colonies at each time point. The branches were each immediately placed into ice-cold sea water to prevent mucus build-up and to slow metabolism, preserving the physiological status of the coral until the sample could be processed, as described below. The time between coral collection and sample processing was always <30 min.

### Temperature perturbation experiment

One to four coral branches were collected from each of ~30 haphazardly selected colonies (no more than one branch per colony was used in each treatment per trial). Branches were attached to micro-centrifuge tubes using underwater epoxy and placed upright on plastic trays. Coral fragments were allowed to recover from collection and mounting and acclimate to the experimental system for at least 3 days prior to beginning the experiment.

The experimental system consisted of nine aquaria, each eight L volume, placed in a large recirculating water bath maintained at 26 °C with an aquarium chiller. Each aquarium was outfitted with a 189 L h^−1^ submersible pump, which provided turbulent water flow, and an aquarium heater, which maintained the temperature of each aquarium at the target level. After the acclimation period, corals were randomly assigned to one of three temperature treatments: 28.1, 28.6, or 29.9 °C (Table 1). Although these corals can tolerate temperatures of 28–28.6 °C for at least several weeks, even a few days of exposure at 29.6–30 °C can induce bleaching (Jokiel & Brown, 2004; Jury & Toonen, 2019). Hence, these treatments span the range from typical summertime temperatures to those which induce rapid bleaching during natural thermal stress events (Jokiel & Brown, 2004). Temperature was spot checked and recorded frequently in each aquarium during each experiment trial. The nine aquaria were arranged in a 3 × 3 grid and treatment temperature assigned to each aquarium in a Latin Square design, to control for row and column effects.

**Table 1:** Table showing treatment seawater temperatures during the thermal stress experiment.

Treatment	Mean (°C)	SEM (°C)	Min (°C)	Max (°C)
28.1	28.09	0.06	27.0	29.1
28.6	28.55	0.05	27.0	29.2
29.9	29.86	0.04	28.9	30.6

**Note:**

Values shown are mean, standard error of the mean (SEM), minimum and maximum temperatures measured with all tanks, experiment trials, and sampling times pooled. Number of observations for each treatment, *n* = 96.

At the beginning of each experimental trial, two randomly selected coral branches were placed into each aquarium (ensuring that not more than one branch per colony was assigned to each treatment). After 24 h, one of the two branches was randomly selected for destructive sampling while the other was destructively sampled after 72 h. Three separate experiment trials to examine symbiont responses were performed during each of September, October, and November, 2006. One additional experiment trial was performed in December, 2006 to assess the potential contribution of coral host NOS activity to that measured for the symbionts.

### Symbiont preparation

Coral tissues containing symbionts were removed from the skeleton using a Waterpik and recirculated, ice-cold artificial sea water (ASW; Coralife scientific grade marine salt, 34.34 g L^−1^). Approximately 400 mL of ASW was used per sample. A portion of the resultant slurry was transferred to a 50 mL centrifuge tube and spun down at 3,100*g* for 5 min at 4 °C, to pelletize the symbionts. The overlaying supernatant containing coral tissue and ASW was decanted and the symbiont pellet was retained. The centrifuge tube was refilled with more coral slurry, the symbiont pellet resuspended, and spun down again. This process was repeated until all symbionts from each sample had been isolated in a single 50 mL centrifuge tube. The pellet was then rinsed 5–8 times by resuspending it in 30 mL ice-cold ASW, centrifuging it at 2,100–3,100*g* for 5 min at 4 °C each time, and decanting so as to remove residual coral tissue. After the final rinse the pellet was resuspended in two mL ice-cold ASW and transferred to a two mL microcentrifuge tube. Six 50 µL aliquots were removed from the homogenized, isolated symbionts and placed into microcentrifuge tubes—five for cell staining and one for symbiont density determination. The remaining symbionts were kept on ice and used for the NOS activity assay, as described below.

Symbiont cell densities were assessed by diluting each 50 µL aliquot with 450 µL ASW and counting four 10-µL subsamples using a hemocytometer. The remaining symbiont isolate in the 50 mL centrifuge tube was then centrifuged to pelletize the symbionts, the supernatant discarded, and the pellet resuspended in ~300–500 µL homogenization buffer (50 mM HEPES, 1 mM EDTA, pH = 7.4, in ASW) to yield a density of 50,000 cells µL^−1^.

### NOS activity assay, symbiont density, and normalization

An aliquot of the symbiont sample at the standardized cell density of 50,000 µL^−1^ was pipetted to a two mL microcentrifuge tube along with glass beads (0.3*g* beads of diameter 425–600 µm and 0.15*g* beads of diameter 150–212 µm, Sigma Aldrich, St. Louis, MS, USA) and homogenized with a Qiagen tissuelyser (Retsch, Haan, Germany) at 30 Hz for 2 min. The sample was centrifuged at 13,000*g* for 2 min to remove the glass beads from the symbiont cell homogenate and the supernatant was pipetted to a 500 µL PCR tube for the NOS assay.

The NOSdetect kit from Stratagene (La Jolla, CA, USA) was used to determine NOS activity following the protocol of Trapido-Rosenthal et al. (2001, 2005. This method relies on the conversion ^3^H-arginine → ^3^H-citruline + NO with subsequent binding and removal of unreacted ^3^H-arginine to assess the rate of NO production *via* NOS, and assuming a 1:1 stoichiometry. A reaction mix containing final concentrations of 0.8 mM CaCl_2_, 1.4 mM NADPH, and 0.028 mCi mL^−1^^3^H-arginine was prepared for each sampling period using the reaction buffer provided in the kit and stock solutions of 6 mM CaCl_2_, 10 mM NADPH, and 1 mCi mL^−1^^3^H-arginine. Subsamples of 14 µL from each symbiont cell homogenate (each containing 700,000 cell equivalents) were transferred to PCR tubes and combined with 36 µL reaction mix. Final concentrations of reagents were 0.6 mM CaCl_2_, 1 mM NADPH, and 0.02 mCi mL^−1^^3^H-arginine. Blanks containing no NOS activity were produced as a procedural control by combining 14 µL homogenization buffer with 36 µL reaction mix. After 1 h incubation each reaction was halted by the addition of 400 µL STOP buffer (100 mM HEPES, 10 mM EDTA, pH = 5.5). Highly vortexed resin from the NOSdetect kit was then added to each tube (100 µL per tube) and the contents were reflux pipetted 20 times to ensure binding of unreacted ^3^H-arginine. The contents were centrifuged at 13,000*g* trapping the ^3^H-arginine bound to the resin in a filter provided in the NOSdetect kit and separating it from the fluid containing ^3^H-citruline. The liquid fraction was transferred to a scintillation vial, combined with five mL Universol^R^ liquid scintillation fluid (MP biomedicals, Solon, OH, USA), capped, shaken vigorously and counts per minute (cpm) read with a Beckman LS 3801 scintillation counter. The blanks were used to provide a baseline correction for the symbiont samples. Counts per minute were converted to attomoles (amol, 10^−18^ mol) ^3^H-citruline symbiont cell^−1^ min^−1^.

The same general procedure was used to assess coral host NOS activity except that coral tissues were removed from the skeleton *via* Waterpik using five mL ice-cold homogenization buffer, rather than ASW, and the NOS assay was performed with a 14 µL aliquot of supernatant containing host tissues rather than freshly isolated symbionts.

Coral skeletal displacement was measured in distilled water in a 100 mL graduated cylinder. Symbiont density in each sample was normalized to skeletal displacement, as was coral host NOS activity (Benayahu & Loya, 1986; Pupier, Bednarz & Ferrier-Pagès, 2018).

### NO production assay

We intended to directly examine rates of NO production using the fluorescent dyes DAF-FM and DAF-FM DA (4-amino-5-methyamino-2′-7′-difluoreoscein diacetate, Molecular Probes, Eugene, OR, USA), which have been used to quantify NO production (Perez & Weis, 2006; Bouchard & Yamasaki, 2008). However, we encountered difficulty with this application. DAF-FM DA is a cell-permeant dye but after entering a cell, native esterases cleave off the diacetate group, making the resultant DAF-FM cell-impermeant and retaining it within the cell. DAF-FM is weakly fluorescent in the absence of NO but reacts with NO to produce a benzotriazole derivative which is 160× more fluorescent than DAF-FM.

After incubation with 9 µM DAF-FM DA or DAF-FM and intact cells or cell homogenates, respectively, fluorescence was read using a SpectraMax M2 96 well plate reader (Molecular Devices). To optimize our fluorescence measurements, during pilot experiments we prepared symbiont isolates and symbiont cell homogenates as described above and examined dilution series of 100–10,000 cells or cell equivalents µL^−1^, using a sample volume of 100 µL. Contrary to expectations, we found that fluorescence (measured as relative fluorescence units, RFU) decreased in proportion to cell or cell equivalent density, suggesting that the symbionts were somehow quenching the fluorescence signal (Fig. S1). Calibration curves of NO production using the NO donor SNAP (S-nitroso-N-acetylpenicillamine, Sigma Aldrich, St. Louis, MO, USA) and mouse isolated NOS (Sigma Aldrich, St. Louis, MO, USA) showed the expected behavior of increasing fluorescence with increasing SNAP concentration using the same solutions and procedure as with the symbionts (Fig. S1). Reactions with mouse NOS were performed using the recommended master mix (final concentrations: 0.4 unit mouse NOS; 1 mM arginine; 12 µM tetrabiodiopterin; 170 µM dithiothreitol; 5 µM oxyhemoglobin; 0.1 mM NADPH; 1 mM magnesium acetate). With mouse NOS, a SNAP concentration of 325 µM produced easily detectable levels of DAF-FM fluorescence, so symbiont samples were incubated with 325 µM SNAP over a cell dilution series to determine the level of detection of NO production using our methods. Unfortunately, rates of NO production in all symbiont isolates were below the level of detection at cell or cell equivalent densities <500 µL^−1^, which were required to reduce symbiont quenching sufficiently to allow any hope of representative fluorescence measurements. As a result, this assay was abandoned during experiment trials. To the best of our knowledge, the apparent fluorescent quenching we observed has not been previously reported, and we advise caution when applying fluorescent techniques to organisms which contain photoactive compounds.

### Symbiont oxidative stress and mortality

We used two cell stains to assess symbiont oxidative stress and cell death in response to high temperature exposure. Fluorescein-derived 2′, 7′-dichlorodihydroflorescein diacetate (H_2_DCFDA, Molecular Probes, Eugene, OR, USA) was used to assess oxidative stress. Like DAF-FM, it is a cell-permeant dye but after entering a cell native esterases cleave off the diacetate group, making the resultant H_2_DCF cell-impermeant and retaining it within the cell. H_2_DCF is essentially non-fluorescent in the absence of oxidants but reacts with hydrogen peroxide (H_2_O_2_) and hydroxyl radicals (·OH) to produce the fluorescent DCF. Symbiont death was assessed with the mortal stain Sytox green (Molecular Probes, Eugene, OR, USA) which is cell-impermeant for live cells but readily enters the compromised membranes of dead cells and stains nucleic acids green. A stock solution of 5 mM H_2_DCFDA was prepared as needed in molecular grade DMSO, which was stored in the dark at −20 °C and was discarded after 2 days. Sytox green (5 mM in DMSO) was allowed to thaw in the dark prior to use.

The five symbiont samples collected from each coral for cell staining were used to produce (1) an unstained negative control, (2) an H_2_DCFDA-stained sample, (3) a Sytox green-stained sample, (4) an H_2_DCFDA-stained positive control, and (5) a Sytox green-stained positive control. The H_2_DCFDA-stained positive control was incubated concurrently with 10 mM exogenous H_2_O_2_, to ensure a highly oxidizing environment was present within the algal cells. For the Sytox green-stained positive control, the symbionts were first killed by placing them in a 55 °C water bath for 10 min. Each sample was then incubated in the dark at a dye concentration of 5 µM (no dye in the negative control) for at least 25 min at room temperature. After incubation, the total symbiont density (stained and unstained) in each sample was determined by counting with a hemacytometer, as described above. The cells were then excited with blue light (excitation 450–490 nm, emission 515–565 nm) to determine the proportion of positively stained cells, using the positive and negative staining controls to set upper and lower bounds, respectively. When imaging the H_2_DCFDA stain, a band pass filter was used to exclude excitation light with a wavelength shorter than 495 nm because chlorophyll autofluorescence interfered with imaging.

### Statistical analysis

For the temperature perturbation experiment, the results of the symbiont NOS activity assay were averaged for each trial, with trial representing a statistical replicate. However, the NOS activity assay could not be completed for several samples during the October, 24 h sampling time, so these data were excluded from the analysis (24 h NOS, *n* = 2; 72 h NOS, *n* = 3). The staining data were analyzed using each nubbin as a statistical replicate. However, the samples for H_2_DCF staining were lost during the September 72 h sampling time (72 h H_2_DCF staining, *n* = 6; all other staining, *n* = 9).

The data were analyzed by two-way ANOVA with temperature and sampling time as factors, followed by a Tukey HSD as a *post hoc* in R v.3.5.2 (R Core Team, 2020). The staining data were arcsine square root transformed prior to analysis.

## Results

### Field investigation

**Figure 1:**
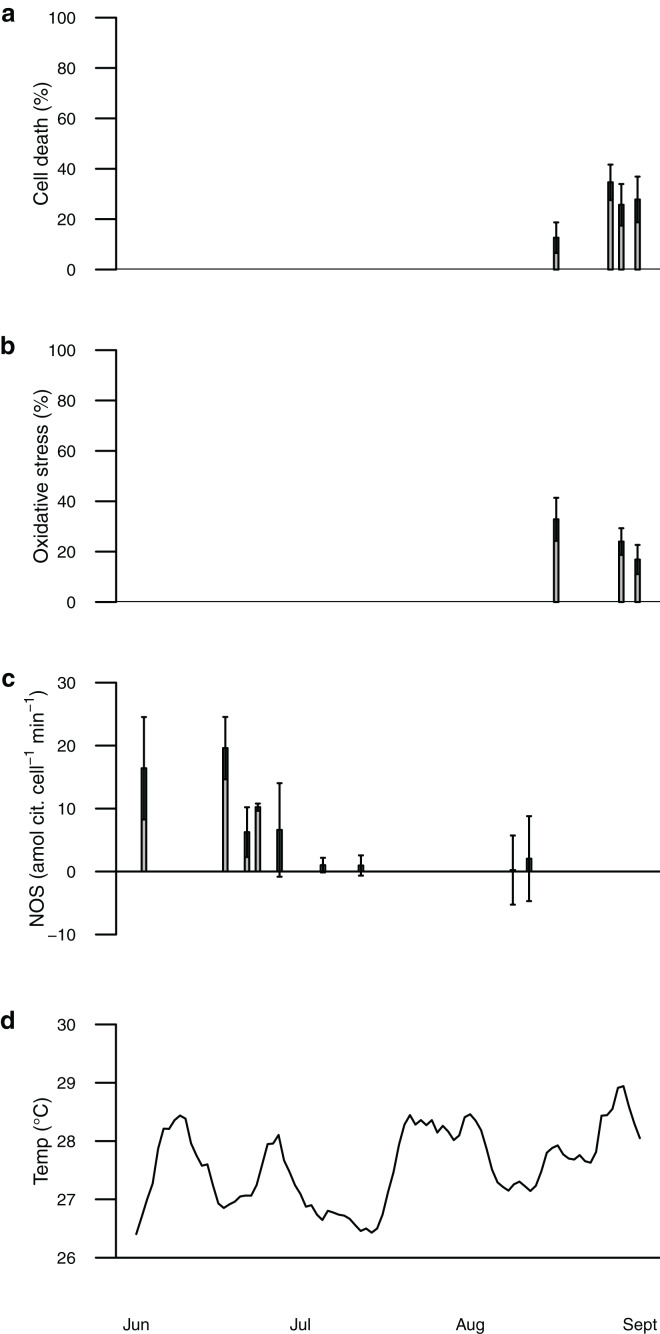
Plot of data from field sampling shown according to sampling date (scale is June 1, 2006–September 1, 2006). Percentage of symbionts from each coral sample positively stained with Sytox green (cell death) shown as black bars in (A), percentage stained with H_2_DCF (oxidative stress) shown as black bars in (B). Staining data only for corals collected mid-Aug to early-Sep. Nitric oxide synthase activity shown as black bars in (C) and temperature shown as black line in (D). NOS activity data for corals collected early-June to mid-August. Bars are mean ± sd, *n* = 1–3 corals per time point.

Seawater temperature recorded by the NOAA weather station at HIMB varied from ~26.5–29 °C during the summer of 2006 (Fig. 1). No coral bleaching was observed at any time during the field study (B Boeing, 2016, personal observations). NOS activity in freshly isolated symbionts ranged from 0.2 to 19.6 amol citrulline cell^−1^ min^−1^, whereas the proportion of oxidatively stressed cells (from H_2_DCF staining) ranged from 13% to 33% and the proportion of dead cells (from Sytox staining) ranged from 13% to 35% (Fig. 1). These values may represent the normal range of symbiont NOS activity, oxidative stress, and cell death in *P. acuta* under typical summertime conditions in Hawai‘i and provide a baseline for our thermal perturbation experiments below.

### Temperature perturbation experiment

Overall symbiont cell density (normalized to coral skeletal displacement) averaged 3.9 ± 2.5 × 10^6^ cells skeletal mL^−1^ and did not differ significantly by treatment. Hence, we successfully sampled the corals during the early stages of heat stress, preceding significant reductions in symbiont density due to bleaching.

NOS activity increased according to temperature treatment as well as time of exposure (Table S1). NOS activity was similar in all treatments to that measured during the field investigation, except for the values measured under the 29.9 °C, 72 h treatment (Table 1), which were substantially higher than those measured in the field. Coral host NOS activity was examined to determine if residual host tissue could have biased the symbiont measurements, but was 3–6 orders of magnitude lower than those from the symbionts (see Supplemental Data File). Alternatively, it is possible that the host fraction contained minor residual contamination from the symbionts and the very low levels of NOS activity measured was not derived from the coral hosts. Regardless, these results gave us confidence that the symbionts were the primary site of NOS activity.

**Figure 2:**
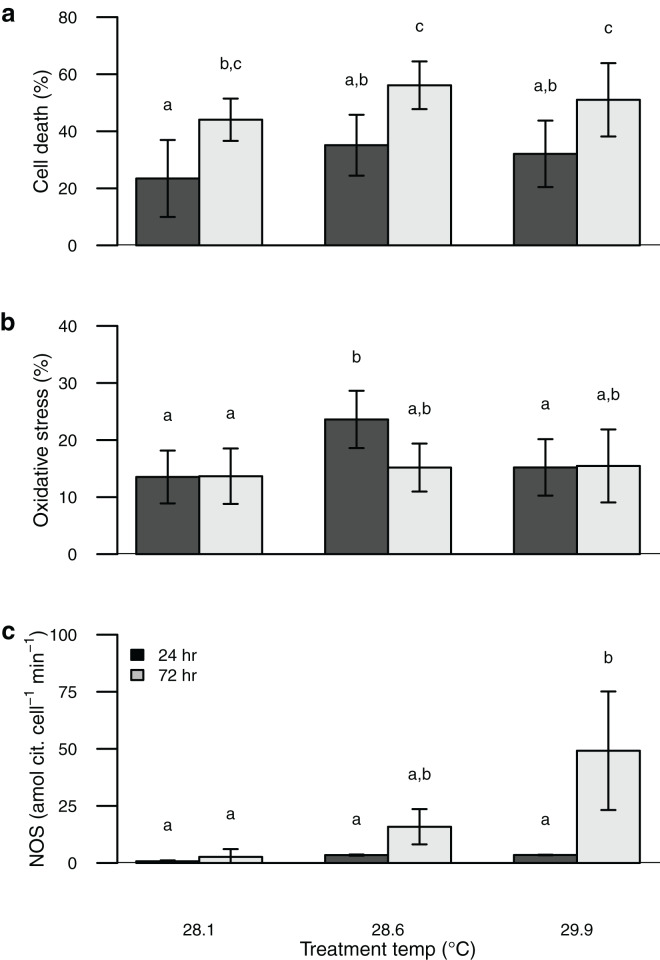
Barplot of data from the thermal stress experiment shown according to temperature treatment (28.1, 28.6, 29.9 °C) and sampling time (24, 72 h). Sytox green staining (cell death) shown in (A) and H_2_DCF staining (oxidative stress) shown in (B). Bars are mean ± sd, *n* = 6–9 coral nubbins per treatment. Nitric oxide synthase activity shown in (C). Bars are mean ± sd, *n* = 2–3 trials per treatment. Groups sharing letters are not statistically different based on Tukey HSD *post hoc* results.

Symbiont oxidative stress increased with temperature, whereas cell death increased with both elevated temperature and time of exposure (Table S1). These values were similar to or somewhat higher than those obtained from the field-collected corals (Figs. 1 and 2). Unlike NOS activity, these two response variables (oxidative stress and cell death) tended to show maximum values in the intermediate temperature treatment (Fig. 2).

## Discussion

Perhaps the most surprising result of this study is the apparent incongruence among symbiont nitric oxide production (inferred from NOS activity), oxidative stress, and cell death. Temperature stress can lead to photosynthetic dysfunction of algal symbionts, which is expected to result in increased oxidative stress and cell death (Downs et al., 2002; Weis, 2008; Díaz-Almeyda et al., 2017; Oakley & Davy, 2018; Wietheger et al., 2018). Rather than a consistent increase in oxidative stress and cell death with increasing temperature and time of exposure as we predicted, both were highest in the intermediate temperature treatment. In contrast, symbiont NOS activity increased consistently with temperature and time of exposure, congruent with the onset of bleaching. Thus the onset of bleaching in this coral appears to be driven at least in part by elevated NO production, rather than by physiological dysfunction of the symbionts. Indeed, Nielsen, Petrou & Gates (2018) found no evidence that oxidative stress plays a major role in bleaching for this species of coral, and Wietheger et al. (2018) showed that symbiont sensitivity to oxidative stress was a poor predictor of thermal sensitivity. Over the short term (24–72 h) a moderate temperature increase of ~0.5 °C elicits a stronger physiological response from these symbionts than a larger temperature increase of ~2 °C (Fig. 2), whereas the larger temperature increase was associated with higher NOS activity and is known to result in bleaching over longer timeframes (Jokiel & Brown, 2004; Jury & Toonen, 2019). Although we attempted to directly measure NO production with the dyes DAF-FM and DAF-FM DA, we encountered problems with this approach due to apparent fluorescent quenching by the symbionts. Alternative techniques have been developed to characterize NO production both *in vivo* and *in vitro* and applying some of these methods to future studies may help to clarify its role in coral bleaching (Goshi, Zhou & He, 2019). Proteomics or reverse transcription polymerase chain reaction (RT-PCR) to characterize expression levels of the NOS protein(s) as well as immunolocalization would further improve our understanding of the mechanisms involved in this response.

Under non-stressful conditions, several cnidarian hosts, rather than the symbionts, including a scleractinian (hard) coral, octocorallian (soft) coral, and an anemone show the capacity to produce NO (Safavi-Hemami et al., 2010). NOS activity in these species, however, was dependent on calcium concentration *in vitro*, suggesting that cellular depolariziation due to calcium influx might induce NO production within these host cells. Such depolarization *via* calcium influx is a common response to certain stressors, including oxidiative stress, both in plants and in animals (Wahl, Koopmann & Ammon, 1998; Nuhkat et al., 2021).

With elevated temperatures, plants often increase NO production (Parankusam et al., 2017). The biochemical mechanisms which underlie this induction are poorly understood, but may include changes in enzyme phosphorylation, calcium channel activity, and H_2_O_2_ concentration, among others. Increased plant NO production under elevated temperature appears to modify the expression of a variety of genes as well as post-translational alterations to the gene products, resulting in improved responses to oxidative and heat stress (Parankusam et al., 2017). Further, Ross (2014) found that symbionts of the octocorallian (soft) coral *Eunicia fusca* showed innate expression of an NOS protein, whereas under heat stress the NOS became conjugated with heat shock protein 90 (Hsp90), which substantially increased NOS activity. Hence, symbionts may increase NO production under warmer conditions as a physiological mechanism to cope with this stressor. NO is also a critical component in the immune response across the tree of life. If symbionts increase NO production as a mechanism to deal with heat stress, corals, other cnidarians, and additional taxa may react to that production as though the symbionts were a pathogen, resulting in expulsion from the host and the bleaching response (Weis, 2008).

Temperature effects on the coral symbiosis are complicated, however, with many aspects of thermal stress events, including rate of warming, duration, magnitude, and variability of temperature, irradiance, and flow, all modifying the likelihood of coral bleaching (Nakamura & van Woesik, 2001; McClanahan et al., 2005; Middlebrook et al., 2010; Schoepf et al., 2015; Page et al., 2019; Fifer et al., 2021). For example, Berkelmans (2002) found that short-term (3–5 d) exposure to temperatures 2–3 °C above local maxima resulted in higher rates of bleaching on the Great Barrier Reef than long-term exposure at lower temperatures, even under equivalent degree heating days. These data underscore the fact that even if symbionts remain functional under temperature stress, bleaching can still be induced by other physiological responses. It should also be noted that the corals in these experiments were already acclimatized to a normal summertime temperature of ~28 °C. Berkelmans & Willis (1999) found that bleaching thresholds for *P. damicornis* on the Great Barrier Reef were 1 °C higher in summer than in winter, suggesting that some corals undergo seasonal temperature acclimatization. Thus, one might expect the patterns we observed to be shifted toward lower absolute temperatures in corals acclimatized to wintertime rather than summertime conditions. In contrast, Barott et al. (2021) worked on other species (*Montipora capitata* and *Porites compressa*) in the same locations and found no change in bleaching response among corals acclimatized to reefs with differing environmental and temperature profile. It remains to be seen how much of the differences in such responses are taxonomic and how much can be explained by variation in experimental conditions and unmeasured or unreported variation in light, flow or temperature that modify bleaching responses of the coral holobiont (McLachlan et al., 2020; Grottoli et al., 2021).

A fundamental question is how organisms maintain physiological function in the face of external stressors. One strategy under acute stress is to reduce metabolic activity until appropriate environmental conditions return—a sit-and-wait approach (Hand & Hardewig, 1996). The low levels of symbiont oxidative stress and cell death observed at the highest temperature here might be explained by such a strategy. Corals are often able to tolerate temperatures well above known bleaching thresholds for short periods of time (Coles, 1975; Barshis et al., 2010, 2013). The precise physiological mechanisms underlying this tolerance are poorly understood, but nonetheless symbiotic corals appear to have mechanisms at their disposal which allow them to tolerate short-term, high temperature exposure. These data suggest that lower NO production under acute temperature stress or reduced NO-sensitivity (either by symbionts or hosts) could provide one mechanism to survive short-term elevated temperatures. If symbionts or hosts in thermally variable environments show lower upregulation of NOS activity or lower NO-sensitivity under temperature stress than those in more stable environments, it could help to explain their persistence in such environments. Likewise, if genetic controls on NOS regulation or NO-sensitivity vary significantly among symbionts or hosts, then individuals that show lower upregulation or sensitivity under elevated temperatures could experience a selective advantage under a warming climate, potentially driving adaptation over time. Over the long-term, elevated temperatures often lead to physiological stress for symbionts and corals, and acclimatization or adaptation in other key physiological traits are likely required to allow them to persist under climate change (Jokiel & Coles, 1990; Dunn et al., 2004; Barshis et al., 2010, 2013).

Coral bleaching mediated by NO production could also be an exaptive byproduct of normal cell signaling which is adaptive under non-stressful conditions. Normally, symbiont NOS activity per cell is low and approximately constant over a range of symbiont densities (Trapido-Rosenthal et al., 2005). An increase in symbiont density within coral tissues should result in a proportional increase in NO production and bleaching (Cunning & Baker, 2013). If corals are able to sense NO concentration, then under normal circumstances they could use it to help regulate symbiont density within their tissues. Under elevated temperatures symbiont NOS activity and NO production increase dramatically, which the corals might perceive as a substantial increase in symbiont density, causing them to purge symbionts from their tissues at high temperatures and ultimately resulting in bleaching. However, the mechanism must be more complex than simply NO sensing, because corals naturally maintain higher symbiont densities in winter than in summer even though NOS activity per symbiont cell is similar under normal winter and summer conditions (Trapido-Rosenthal et al., 2005) meaning that additional mechanisms must also be involved in regulating symbiont density within coral tissues.

In some previous studies aposymbiotic anemones or cultured symbionts failed to increase NO production under elevated temperatures, suggesting that symbiosis may be critical to initiate this response in some cases (Trapido-Rosenthal et al., 2001, 2005; Perez & Weis, 2006). In contrast, Bouchard and Yamasaki were able to induce increased NO production in cultured symbionts under acute temperature stress (Bouchard & Yamasaki, 2008). Some studies have concluded that the cnidarian host cells are the primary NO source (Perez & Weis, 2006; Safavi-Hemami et al., 2010) whereas others have found the symbiont cells to be the primary NO source (Trapido-Rosenthal et al., 2001, 2005; Bouchard & Yamasaki, 2008). Both partners appear capable of producing NO in response to elevated temperatures, but only one of the two seems to dominate the response in a particular symbiotic association. The reasons for this disparity are unknown, but suggest that cnidarian bleaching can be driven by physiological responses originating in either the host or the symbiont cells. A rigorous explanation of why cnidarian hosts or algal symbionts increase their NOS activity in response to elevated temperature in some cases and not in others would improve our understanding of the mechanism of cnidarian bleaching and may help to explain variation in bleaching susceptibility both within and among species (van Oppen & Lough, 2018). Here we show that symbionts freshly isolated from the coral *Pocillopora acuta* exhibit NOS activity and that NOS activity increases with both temperature and time of exposure. In contrast, symbiont oxidative stress and cell death were highest under intermediate rather than maximal temperature, suggesting that NOS activity, rather than symbiont dysfunction, may be the more important driver of coral bleaching under acute temperature stress. Our study adds to a growing body of literature showing that elevated NO production, independent of stress or damage incurred by the symbionts, is an important mediator of coral bleaching under elevated temperatures.

## Supplemental Information

10.7717/peerj.13321/supp-1 Supplemental Information 1 Plot of pilot experiments examining NO production with the NO-indicator DAF-FM. Calibration curve using mouse NOS and increasing concentrations of the NO-donor SNAP shown in (a). DAF-FM fluorescence examined over a symbiont dilution series of 100–10,000 cells µL^−1^, with (open symbols) or without (closed symbols) 325 µM SNAP shown in (b). Data expressed in relative fluorescence units (RFU). Sample size, *n* = 3 for each dataset. Where error bars and symbols are not evident in (b) it is because they are smaller than the symbol size, or symbols are overlapping.

10.7717/peerj.13321/supp-2 Supplemental Information 2 ANOVA test results from the thermal stress experiment. Df = degrees of freedom, Sum Sq = sum of squares, Mean Sq = mean of squares, F = F-value, and p = *p*-value. *P*-values shown in bold are significant at alpha = 0.05.

10.7717/peerj.13321/supp-3 Supplemental Information 3 Raw data.

## Additional Information and Declarations

### Data Availability

The following information was supplied regarding data availability: The raw data are available in the Supplemental File.
