# Supplementary figures and images for: Nitric oxide production rather than oxidative stress and cell death is associated with the onset of coral bleaching in Pocillopora acuta
Source: PeerJ. 2022 Jun 1;10:e13321. doi: 10.7717/peerj.13321 (PMC9166681; doi:10.7717/peerj.13321)

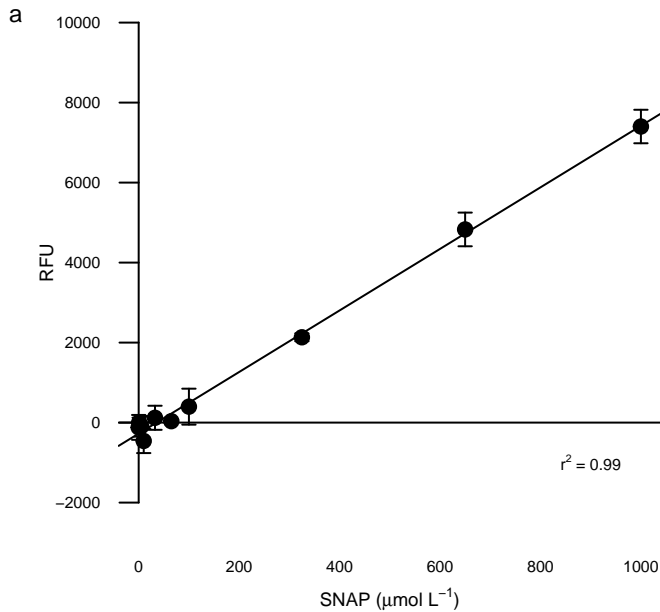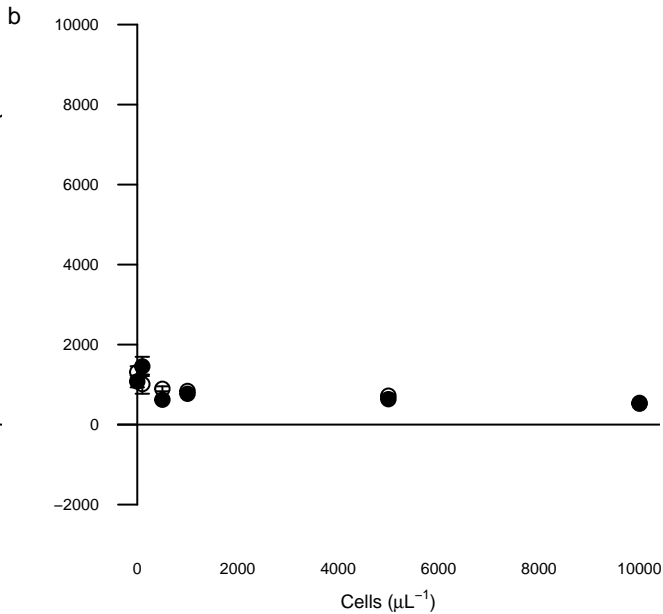

Supplement: Supplemental Information 1 — Calibration curve using mouse NOS and increasing concentrations of the NO-donor SNAP shown in (a). DAF-FM fluorescence examined over a symbiont dilution series of 100–10,000 cells µL−1, with (open symbols) or without (closed symbols) 325 µM SNAP shown in (b). Data expressed in relative fluorescence units (RFU). Sample size, n = 3 for each dataset. Where error bars and symbols are not evident in (b) it is because they are smaller than the symbol size, or symbols are overlapping. [file peerj-10-13321-s001.pdf]
