# Supplementary material for: Nitric oxide production rather than oxidative stress and cell death is associated with the onset of coral bleaching in Pocillopora acuta
Source: PeerJ. 2022 Jun 1;10:e13321. doi: 10.7717/peerj.13321 (PMC9166681; doi:10.7717/peerj.13321)
Supplement: Supplemental Information 2 — Df = degrees of freedom, Sum Sq = sum of squares, Mean Sq = mean of squares, F = F-value, and p = p-value. P-values shown in bold are significant at alpha = 0.05. [file peerj-10-13321-s002.docx]

**Table S1**. ANOVA test results from the thermal stress experiment. Df=degrees of freedom, Sum Sq=sum of squares, Mean Sq=mean of squares, F=F-value, and p=p-value. P-values shown in bold are significant at alpha=0.05.

| Response | Df | Sum Sq | Mean Sq | F | p |
| --- | --- | --- | --- | --- | --- |
| NOS |  |  |  |  |  |
| Temp | 2 | 2205 | 1102.4 | 6.650 | **0.017** |
| Time | 1 | 1440 | 1440.2 | 8.687 | **0.016** |
| Temp×Time | 2 | 1253 | 626.6 | 3.780 | 0.064 |
| Residuals | 9 | 1492 | 165.8 |  |  |
|  |  |  |  |  |  |
| Cell death |  |  |  |  |  |
| Temp | 2 | 0.1655 | 0.0827 | 5.472 | **<0.01** |
| Time | 1 | 0.6457 | 0.6457 | 47.701 | **<0.001** |
| Temp×Time | 2 | 0.0033 | 0.0017 | 0.110 | 0.896 |
| Residuals | 48 | 0.7258 | 0.0151 |  |  |
|  |  |  |  |  |  |
| Oxid. stress |  |  |  |  |  |
| Temp | 2 | 0.06419 | 0.03210 | 6.460 | **<0.01** |
| Time | 1 | 0.01417 | 0.01417 | 2.851 | 0.099 |
| Temp×Time | 2 | 0.02816 | 0.01408 | 2.834 | 0.071 |
| Residuals | 39 | 0.19376 | 0.00497 |  |  |
